# Ultrasound-Guided Pleural Effusion Drainage: Effect on Oxygenation, Respiratory Mechanics, and Liberation from Mechanical Ventilation in Surgical Intensive Care Unit Patients

**DOI:** 10.3390/diagnostics11112000

**Published:** 2021-10-28

**Authors:** Hsin-Yueh Fang, Ko-Wei Chang, Yin-Kai Chao

**Affiliations:** 1 Division of Thoracic Surgery, Chang Gung Memorial Hospital, Taoyuan 333, Taiwan; leomoon0723@gmail.com; 2 Department of Thoracic Medicine, Chang Gung Memorial Hospital, Taoyuan 333, Taiwan; b9302072@cgmh.org.tw

**Keywords:** post-operative pleural effusion, ultrasound, drainage, oxygenation, lung mechanics

## Abstract

The question as to whether an aggressive management of post-operative pleural effusion may improve clinical outcomes after major surgery remains unanswered. The aim of this study was to investigate the effect of ultrasound-guided pleural effusion drainage on oxygenation, respiratory mechanics, and liberation from mechanical ventilation in surgical intensive care unit patients. Oxygenation and respiratory mechanics were measured before and after drainage. Over an 18-month period, a total of 62 patients were analyzed. The mean drainage volume during the first 24 h was 864 ± 493 mL, and there were no procedural complications. Both the mean PaO_2_/FiO_2_ ratio and lung compliance improved after drainage. Additionally, 41.9% (*n* = 26) of patients were ventilator-free within 72 h after drainage. Multivariable logistic regression analysis revealed that non-cardiovascular or thoracic surgery (odds ratio [OR] = 4.968, *p* = 0.046), a longer time interval from operation to the onset of pleural effusion (OR = 1.165, *p* = 0.005), and a higher peak airway pressure (OR = 1.303, *p* = 0.009) were independent adverse predictors for being free from mechanical ventilation within 72 h after drainage. Specifically, patients with a time from surgery to the onset of pleural effusion ≤6 days—but not those with an interval >6 days—showed a significant post-procedural improvement in terms of PaO_2_/FiO_2_ ratio, PaCO_2_, peak airway pressure, and dynamic lung compliance. In summary, ultrasound-guided pleural effusion drainage resulted in significant clinical benefits in mechanically ventilated ICU patients after major surgery—especially in those with early-onset effusion who received thoracic surgery.

## 1. Introduction

Pleural effusion—defined as the accumulation of fluid in the pleural space resulting from an imbalance between production and resorption [1,2]—is a common complication of major surgery [3,4,5,6]. In general, post-operative pleural effusions are self-limiting and do not require specific treatment [3,4]. Nonetheless, the question as to whether an aggressive management of post-operative pleural effusion may improve the clinical outcomes of critically ill patients who require post-operative mechanical ventilation (MV) remains unanswered. In this scenario, this study was undertaken to investigate the effects of ultrasound-guided pleural effusion drainage on oxygenation and respiratory mechanics in mechanically ventilated surgical intensive care unit (ICU) patients. Additionally, we applied multivariable logistic regression analysis to identify the independent predictors of liberation from MV within the first 72 h from ultrasound-guided pleural effusion drainage [7].

## 2. Materials and Methods

### 2.1. Study Patients

This retrospective study, using data from patients who were admitted to an ICU after major surgery between November 2019 and April 2021, was conducted in accordance with the Declaration of Helsinki. The eligibility criterion was the requirement for pleural effusion drainage while the patient was under mechanical ventilation. When two or more drainages were necessary, only the first procedure was included in the analysis. Oxygenation and respiratory mechanics were measured before and after drainage. Liberation from MV within the first 72 post-procedural hours [7] was the main outcome of interest. The study protocol was approved by the local Ethics Committee (CGMHIRB-202100904B0). The requirement for written patient informed consent was waived due to the study design.

### 2.2. Mechanical Ventilation, Pleural Drainage, and Physiological Measurement

MV settings were thoroughly adjusted by ICU respiratory therapists according to the results of arterial blood gas analysis and clinical conditions. A pressure control mode was used for all patients. Following consultation with ICU physicians, thoracic surgeons were in charge of all ultrasound-guided pleural effusion drainage procedures. A small-bore catheter (Fr 10 or Fr 14) was positioned under ultrasound guidance by a thoracic surgeon using the Seldinger’s technique.

Arterial blood gas analysis was routinely performed at 1:00 a.m., when we concomitantly recorded data on MV settings and lung mechanics. Respiratory and hemodynamic parameters were collected at the following time points: one day before ultrasound-guided pleural effusion drainage (T −1); on the procedural day (T 0), and one day thereafter (T +1). The variables of interest included the PaO_2_/FiO_2_ ratio, PaCO_2_, pH, peak airway pressure, positive end expiratory pressure (PEEP), tidal volume/predicted body weight, respiratory rate, dynamic driving pressure, and dynamic lung compliance. The variations observed from T −1 to T 0 were considered as baseline changes and served as reference for further comparisons. The changes occurring between T 0 and T +1 were regarded as the consequence of ultrasound-guided pleural effusion drainage. The dynamic driving pressure was defined as the peak airway pressure minus PEEP, whereas the dynamic compliance was calculated as the tidal volume divided by the dynamic driving pressure. The chest plain film was inspected at least once every 2 days in all participants. The onset of pleural effusion was established after a thorough review of all chest plain films.

### 2.3. Data Collection and Statistical Analysis

The sequential organ failure assessment (SOFA) score [8] served as a proxy for disease severity. Data on laboratory parameters and SOFA scores were collected within 3 pre-procedural days. Simultaneous bilateral pleural effusion drainage was considered as a single procedure for the purpose of analysis.

Continuous variables are presented as means ± standard, and intergroup comparisons were performed using the Student’s *t*-test or the Mann–Whitney *U* test (as appropriate). Paired continuous data were analyzed using the Student’s *t*-test or the McNemar’s test according to the underlying distribution (Gaussian *versus* skewed, respectively). The optimal cutoff values for continuous variables were determined with the Youden’s index from receiver operating characteristic (ROC) curve analysis. Groups were compared on categorical data by Pearson’s chi-square or Fisher’s exact test (as appropriate). A multivariable logistic regression model was applied to identify the independent predictors of liberation from MV, with a threshold of *p* < 0.10 in univariate analysis for inclusion in the final model. All calculations were carried out in SPSS, version 22.0.0 (IBM, Armonk, NY, USA). Statistical significance was determined by a two-tailed *p* value < 0.05.

## 3. Results

### 3.1. Patient Characteristics

**Figure 1:**
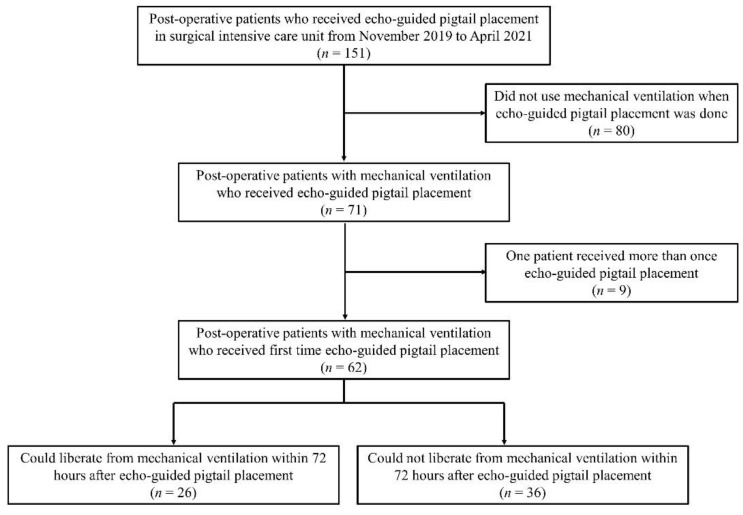
Flow of patients through the study.

A study flowchart is provided in Figure 1. The general characteristics of the study participants are summarized in Table 1. Most patients were men (71.0%) and the mean age of the sample was 61.4 ± 13.4 years. The most common surgical type was abdominal surgery (51.6%) followed by thoracic and cardiovascular surgery (35.5%). The mean time interval from the index operation to ultrasound-guided pleural effusion drainage was 10.8 ± 11.9 days. Fourteen patients received pleural effusion drainage within 1 day of the operation, whereas four underwent the procedure more than 1 month after surgery.

### 3.2. Procedural Outcomes

A total of 62 ultrasound-guided pleural effusion drainages were analyzed in this study. The mean drainage volume during the first day and the mean duration of pleural effusion drainage were 864 ± 493 mL and 18.3 ± 12.9 days, respectively. Only one case of post-procedural pneumothorax was observed on chest imaging, whereas there were no major bleeding episodes.

### 3.3. Changes in Arterial Blood Gas Parameters and Respiratory Mechanics before and after the Procedure

No significant differences were observed with respect to oxygenation and respiratory mechanics between T −1 and T 0 (Table 2). On analyzing changes that occurred from T 0 to T +1, the following modifications were observed: increase in the PaO_2_/FiO_2_ ratio (from 280 ± 102 to 319 ± 101 mmHg, *p* = 0.002), reduction in peak airway pressure (from 23 ± 5 to 22 ± 6 cmH_2_O, *p* = 0.01), reduction in dynamic driving pressure (from 14 ± 5 to 13 ± 5 cmH_2_O, *p* = 0.008), and increase in dynamic lung compliance (from 40 ± 18 to 46 ± 21 mL/cmH_2_O, *p* = 0.003; Table 2).

### 3.4. Frequency and Predictors of Freedom from Mechanical Ventilation within the First 72 Post-Procedural Hours

Liberation from MV within the first 72 post-procedural hours was achieved in 26 patients. In univariate logistic regression analysis, we identified the following adverse predictors for being free from mechanical ventilation within 72 h: non-cardiovascular or thoracic surgery (odds ratio [OR] = 3.00, *p* = 0.046), a longer time interval from operation to the onset of pleural effusion (OR = 1.10, *p* = 0.013), and a higher peak airway pressure (OR = 1.17, *p* = 0.004; Table 3). The time interval from the onset of pleural effusion to pleural effusion drainage was not retained in the model as a significant predictor. After adjustment for potential confounders, multivariable logistic regression analysis revealed that non-cardiovascular or thoracic surgery (OR = 4.97, *p* = 0.046), a longer time interval from operation to the onset of pleural effusion (OR = 1.16, *p* = 0.005), and a higher peak airway pressure (OR = 1.30, *p* = 0.009) were independent adverse predictors for being free from mechanical ventilation within 72 h (Table 3).

**Figure 2:**
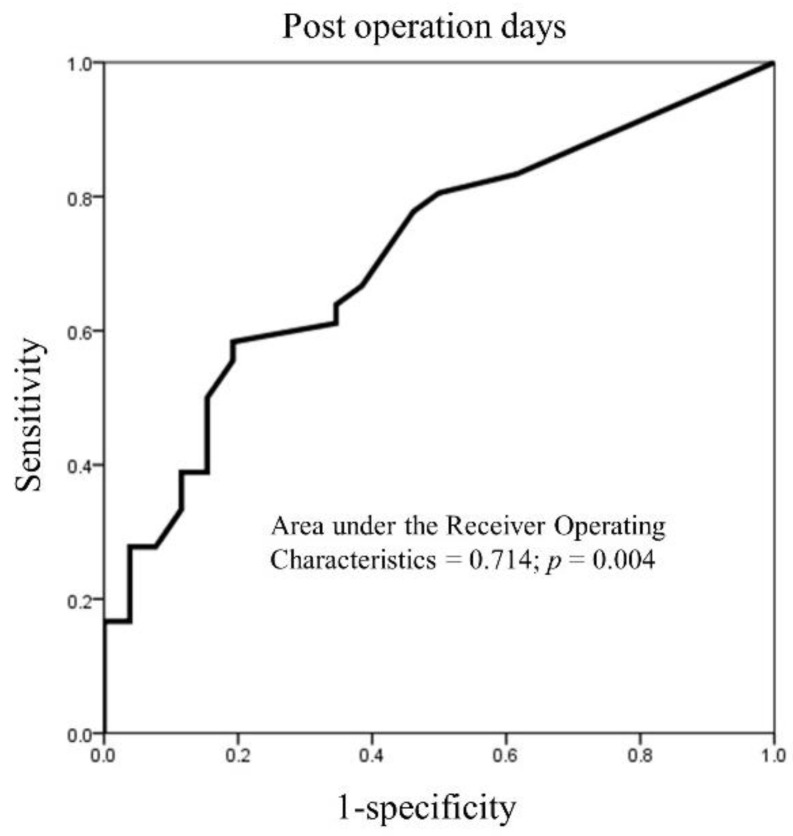
Receiver operating characteristic curve to identify the optimal cutoff value for the time interval from surgery to pleural effusion drainage using liberation from mechanical ventilation within the first 72 post-procedural hours as the outcome of interest.

We subsequently applied ROC curve analysis to identify the optimal cutoff value for the time interval from surgery to the onset of pleural effusion using liberation from MV within the first 72 post-procedural hours as the outcome of interest. The results revealed a C-statistic of 0.71 (95% confidence interval, 0.59–0.84, *p* = 0.004; Figure 2). The optimal cutoff point for the interval from surgery to the onset of pleural effusion was 6.5 days (corresponding Youden’s index: 0.391). The study patients were therefore divided into two groups according the time elapsed from surgery to the onset of pleural effusion, as follows: interval ≤6 days (*n* = 36) and interval >6 days (*n* = 26).

### 3.5. Subgroup Analyses According to the Time Interval from Surgery to Pleural Effusion Drainage

**Figure 3:**
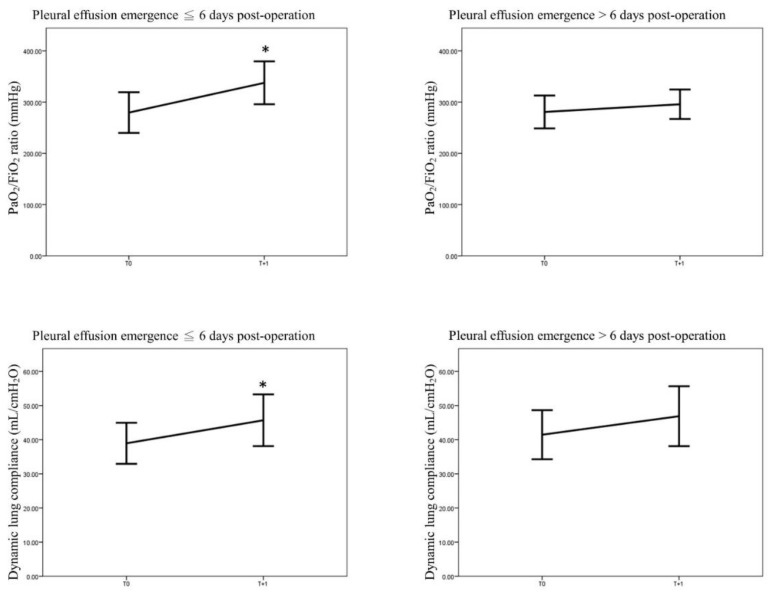
Changes in the PaO_2_/FiO_2_ ratio and lung compliance in patients stratified according to the time interval from the onset of pleural effusion to pleural effusion drainage (≤6 days versus >6 days); * *p* value < 0.05.

On analyzing patients with a time from surgery to the onset of pleural effusion >6 days, no significant changes in the PaO_2_/FiO_2_ ratio and lung compliance were observed from T 0 to T +1. However, the following modifications were observed from T 0 to T +1 in patients with a time from surgery to the onset of pleural effusion ≤ 6 days: increase in the PaO_2_/FiO_2_ ratio (from 278 ± 122 to 338 ± 118 mmHg, *p* = 0.006), reduction in peak airway pressure (from 23 ± 5 to 21 ± 5 cmH_2_O, *p* = 0.002), reduction in dynamic driving pressure (from 15 ± 5 to 13 ± 4 cmH_2_O, *p* = 0.002) and increase in lung compliance (from 39 ± 18 to 46 ± 21 mL/ cmH_2_O, *p* = 0.007; Table 4 and Figure 3).

## 4. Discussion

The results of this retrospective study demonstrate that ultrasound-guided pleural effusion drainage is safe and effective in mechanically ventilated surgical ICU patients—in whom significant post-procedural improvements were observed both in terms of oxygenation (PaO_2_/FiO_2_ ratio) and respiratory mechanics (lung compliance). Our investigation has several strengths. First, this is, to our knowledge, the largest study to date to focus on the value of an aggressive pleural effusion management in surgical ICU patients. Second, we were able to identify a specific patient subgroup which was more likely to achieve freedom from MV within the first 72 post-procedural hours. Specifically, only patients with a time from surgery to the onset of pleural effusion ≤67 days benefitted from ultrasound-guided pleural effusion drainage. Conversely, those with a time from surgery to the onset of pleural effusion >67 days did not show appreciable improvements with respect to both oxygenation and respiratory mechanics.

While a positive impact of pleural effusion drainage on patient oxygenation has been reported in several previous investigations [9,10,11,12,13], the consequences of this procedure on respiratory mechanics are a matter of ongoing debate and deserve an in-depth discussion. Talmer et al. [9] observed a significant increase in the dynamic compliance following thoracocentesis (from 27 to 36 mL/mmHg) without appreciable changes in peak airway pressure. While Razazi et al. [12] showed significant post-procedural changes in both plateau pressure (from 20 to 18 mmHg) and respiratory system compliance (from 32 to 36 mL/mmHg), two independent studies [14,15] did not observe significant changes in lung compliance. On analyzing the post-procedural changes in our surgical ICU patients, we found a significant decrease in peak airway pressure (from 23 to 22 mmHg), a non-significant increase in tidal volume/predicted body weight, and a significant increase in dynamic lung compliance (from 40 to 46 mL/mmHg). One potential explanation for the apparent discrepancies with the published literature is that our patients were intubated before surgery in the absence of any pre-existing lung abnormality. The main underlying reason for the decreased lung compliance observed in our study was the development of pleural effusion and—in this scenario—drainage was expected to provide clinical benefits.

Another important finding from this study is that the time from surgery to the onset of pleural effusion was an independent predictor of freedom from MV within the first 72 post-procedural hours. Additionally, patients with a time from surgery to pleural effusion drainage ≤6 days—but not those with an interval >6 days—showed a significant post-procedural improvement in terms of PaO_2_/FiO_2_ ratio, PaCO_2_, peak airway pressure, and dynamic lung compliance. A potential explanation for such differences may lie in the different etiologies of early *versus* late pleural effusions. Among patients who had undergone cardiac surgery, early pleural effusion is generally caused by pleural [16] or left phrenic nerve [17] injuries; conversely, late effusions are most commonly due to pericarditis [5], pleural inflammation [18], or post-cardiac injury syndrome [19]. Notably, prolonged mechanical ventilation is a recognized risk factor for ventilator-acquired pneumonia after cardiac surgery [20]. Additionally, the presence of pleural effusion may result in the failure to discontinue MV [21] and long-term ventilator dependence is characterized by a high frequency of muscle weakness [22], heart failure [23], and nutritional problems [24]. Our study did not include a control group of ICU surgical patients who did not undergo pleural effusion drainage; therefore, we cannot conclude that this procedure was futile for cases with a time from surgery to the onset of pleural effusion >6 days. However, these patients failed to show significant post-procedural improvement in terms of PaO_2_/FiO_2_ ratio and dynamic lung compliance. Collectively, these results suggest that the implementation of ultrasound-guided pleural effusion drainage in this patient group should be carefully weighed, especially in high-risk situations (e.g., presence of a bleeding diathesis).

Several limitations of our study merit comment. First, the sample size was not sufficiently large to stratify patients according to different surgical types and, for that reason, larger prospective cohorts are needed. However, all participants were mechanically ventilated when they underwent ultrasound-guided pleural effusion drainage and a significant number of patients who did not require MV were not eligible for inclusion. Second, pleural fluid cultures were not systematically performed in all participants; however, 83.0% of pleural fluid samples were subjected to microbiological analysis and positivity was detected in one case only. Taken together, these results suggest that infections were not a leading cause of pleural effusions in our study. Third, we do not have data for patients who showed limited pleural effusions and, consequently, did not undergo drainage. We are aware that a comparison of clinical outcomes for patients with different amounts of pleural effusion would likely be prone to bias. Nonetheless, the decision to perform ultrasound-guided pleural effusion drainage should primarily be guided by clinical reasons (e.g., presence of respiratory failure). A properly designed randomized controlled study will be required to identify a suitable comparator for patients who had undergone pleural effusion drainage.

## 5. Conclusions

Ultrasound-guided pleural effusion drainage is safe and effective in mechanically ventilated surgical ICU patients—among whom it produced significant post-procedural improvements both in terms of oxygenation and dynamic lung compliance. Patients with a time from surgery to the onset of pleural effusion ≤6 days were more likely to benefit from this procedure.

## Figures and Tables

**Table 1:** General characteristics of the study patients.

	Total Patients
	(*n* = 62)
Age (years)	61.4 ± 13.4
Sex (Men/women)	44/18
Body mass index (kg/m^2^)	24.1 ± 4.2
Opeartion type	
Cardiovascular or thoracic	22 (35.5%)
Abdomen	32 (51.6%)
Burn	2 (3.2%)
Brain	1 (1.6%)
Limbs	3 (4.8%)
Neck	2 (3.2%)
Side	
Left	22 (35.5%)
Right	37 (59.7%)
Bilateral	3 (4.8%)
SOFA score	8.4 ± 3.8
Laboratory parameters	
White blood cells (1000/μL)	12.9 ± 6.5
Platelets (1000/μL)	160 ± 119
Prothrombin time/international normalized ratio	1.2 ± 0.4
Total bilirubin (mg/dL)	4.1 ± 6.5
Creatinine (mg/dL)	2.2 ± 2.3
Arterial blood gas analysis before pleural effusion drainage	
PaO_2_/FiO_2_ ratio (mmHg)	280 ± 102
PaCO_2_ (mmHg)	38 ± 7
pH	7.4 ± 0.1
Ventilator settings before pleural effusion drainage	
Peak airway pressure (cmH_2_O)	23 ± 5
Positive end expiratory pressure (cmH_2_O)	8 ± 1
Tidal volume/predicted body weight (mL/Kg)	9 ± 2
Respiratory rate (/minute)	19 ± 4
Dynamic driving pressure (cmH_2_O)	14 ± 5
Dynamic lung compliance (mL/cmH_2_O)	40 ± 18
Time interval from operation to the onset of pleural effusion (days)	9 ± 11
Time interval from the onset of pleural effusion to pleural effusion drainage (days)	1.7 ± 1.8
Chest ultrasound findings	
Fluid height (number of intercostal spaces)	3.5 ± 1.3
Fluid depth (cm)	4.3 ± 1.6
Inadequate diaphragm movements	10 (16.1%)
Drainage volume (mL)	
During the first day	864 ± 493
Average per day	233 ± 168

**Table 2:** Arterial blood gas analyses and respiratory mechanics before and after ultrasound-guided pleural effusion drainage.

	T −1	T 0	T +1	*p* Value (T −1 vs. T 0)	*p* Value (T 0 vs. T +1)
PaO_2_/FiO_2_ ratio (mmHg)	300 ± 146	280 ± 102	319 ± 101	0.92	0.002 *
PaCO_2_ (mmHg)	38 ± 6	38 ± 7	37 ± 7	0.79	0.14
pH	7 ± 0.1	7 ± 0.1	7 ± 0.1	0.42	0.13
Peak airway pressure (cmH_2_O)	23 ± 5	23 ± 5	22 ± 6	0.13	0.01 *
Positive end expiratory pressure (cmH_2_O)	9 ± 1	8 ± 1	8 ± 1	0.78	0.74
Tidal volume/predict body weight (mL/Kg)	9 ± 2	9 ± 2	9 ± 2	0.20	0.35
Respiratory rate (per min)	18 ± 4	18 ± 4	18 ± 4	0.59	0.26
Dynamic driving pressure (cmH_2_O)	15 ± 4	14 ± 5	13 ± 5	0.10	0.008 *
Dynamic lung compliance (mL/cmH_2_O)	37 ± 12	40 ± 18	46 ± 21	0.22	0.003 *

Abbreviations: T −1: 1 day before ultrasound-guided pleural effusion drainage; T 0: day of ultrasound-guided pleural effusion drainage; T 1: 1 day after ultrasound-guided pleural effusion drainage. * *p* < 0.05

**Table 3:** Univariate and multivariable logistic regression analysis of adverse predictors for being free from mechanical ventilation within 72 h after pleural effusion drainage.

	Univariate Analysis		Multivariable Analysis	
	Odds Ratio (95% CI)	*p*	Odds Ratio (95% CI)	*p*
Sex				
Woman	1 (reference)			
Man	0.8 (0.3–2.6)	0.75		
Age, per 1 year increment	1.01 (0.97–1.05)	0.51		
Body mass index, per 1 kg/m^2^ increament	1.08 (0.95–1.23)	0.22		
Operation type				
Cardiovascular or thoracic	1 (reference)		1 (reference)	
Others	3.00 (1.02–8.80)	0.046 *	4.97 (1.03–24.04)	0.046 *
SOFA score, per 1 increment	1.09 (0.95–1.25)	0.24		
Time interval from operation to the onset of pleural effusion, per 1 day increment	1.10 (1.02–1.19)	0.013 *	1.16 (1.05–1.30)	0.005 *
Time interval from the onset of pleural effusion to pleural effusion drainage, per 1 day increment	1.5 (0.90–1.74)	0.19		
Average pleural effusion drainage volume per day, per 1 mL increament	0.999 (0.996–1.002)	0.63		
Laboratory parameters				
PT/INR, per 1 increament	1.08 (0.28–4.19)	0.91		
Blood gas analyses and respiratory mechanics before pleural effusion drainage				
Positive end expiratory pressure, per 1 cm H_2_O increment	1.47 (0.97–2.23)	0.07	1.36 (0.65–2.86)	0.42
Peak airway pressure, per 1 cm H_2_O increment	1.17 (1.05–1.31)	0.004 *	1.30 (1.07–1.59)	0.009 *
Inadequate diaphragm movements				
No	1 (reference)		1 (reference)	
Yes	8.33 (0.98–70.57)	0.05	13.44 (0.59–304.68)	0.10

Abbreviations: CI, confidence interval; SOFA, sequential organ failure assessment; PT/INR, prothrombin time/international normalized ratio. * *p* < 0.05

**Table 4:** Changes in arterial blood gas parameters and respiratory mechanics before and after ultrasound-guided pleural effusion drainage in patients with the time interval from the onset of pleural effusion to pleural effusion drainage ≤6 days or >6 days.

	≤6 Days (*n* = 36)			>6 Days (*n* = 26)		
	T 0	T +1	*p* Value	T 0	T +1	*p* Value
PaO_2_/FiO_2_ ratio (mmHg)	278 ± 122	338 ± 118	0.006 *	281 ± 79	296 ± 71	0.22
PaCO_2_ (mmHg)	39 ± 8	36 ± 7	0.07	38 ± 5	38 ± 7	0.46
pH	7 ± 0.1	7 ± 0.1	0.04 *	7 ± 0.1	7 ± 0.1	0.32
Peak airway pressure (cmH_2_O)	23 ± 5	21 ± 5	0.002 *	23 ± 6	22 ± 7	0.81
Positive end expiratory pressure (cmH_2_O)	8 ± 1	8 ± 1	0.66	9 ± 2	9 ± 2	>0.99
Tidal volume/predict body weight (mL/Kg)	9 ± 2	9 ± 2	0.80	9 ± 1	9 ± 1	0.26
Respiratory rate (per min)	19 ± 5	17 ± 3	0.04 *	18 ± 3	19 ± 5	0.51
Dynamic driving pressure (cmH_2_O)	15 ± 5	13 ± 4	0.002 *	14 ± 5	14 ± 6	0.81
Dynamic lung compliance (mL/ cmH_2_O)	39 ± 18	46 ± 21	0.007 *	41 ± 18	47 ± 22	0.17

Abbreviations: T 0, procedural day; T +1, one day after ultrasound-guided pleural effusion drainage. * *p* < 0.05

## Data Availability

The datasets used and/or analyzed during the current study are available from the corresponding author upon reasonable request.
